# Enzymes for production of whey protein hydrolysates and other value-added products

**DOI:** 10.1007/s00253-024-13117-2

**Published:** 2024-05-31

**Authors:** José Matías Irazoqui, Gonzalo Manuel Santiago, María Esperanza Mainez, Ariel Fernando Amadio, María Florencia Eberhardt

**Affiliations:** Instituto de Investigación de La Cadena Láctea (CONICET-INTA), 2300 Rafaela, Argentina

**Keywords:** Enzymes, Whey proteases, Biopeptides, β-galactosidases, Galactooligosaccharides

## Abstract

**Abstract:**

Whey is a byproduct of dairy industries, the aqueous portion which separates from cheese during the coagulation of milk. It represents approximately 85–95% of milk’s volume and retains much of its nutrients, including functional proteins and peptides, lipids, lactose, minerals, and vitamins. Due to its composition, mainly proteins and lactose, it can be considered a raw material for value-added products. Whey-derived products are often used to supplement food, as they have shown several physiological effects on the body. Whey protein hydrolysates are reported to have different activities, including antihypertensive, antioxidant, antithrombotic, opioid, antimicrobial, cytomodulatory, and immuno-modulatory. On the other hand, galactooligosaccharides obtained from lactose can be used as prebiotic for beneficial microorganisms for the human gastrointestinal tract. All these compounds can be obtained through physicochemical, microbial, or enzymatic treatments. Particularly, enzymatic processes have the advantage of being highly selective, more stable than chemical transformations, and less polluting, making that the global enzyme market grow at accelerated rates. The sources and different products associated with the most used enzymes are particularly highlighted in this review. Moreover, we discuss metagenomics as a tool to identify novel proteolytic enzymes, from both cultivable and uncultivable microorganisms, which are expected to have new interesting activities. Finally enzymes for the transformation of whey sugar are reviewed. In this sense, carbozymes with ß-galactosidase activity are capable of lactose hydrolysis, to obtain free monomers, and transgalactosylation for prebiotics production.

**Key points:**

• *Whey can be used to obtain value-added products efficiently through enzymatic treatments*

• *Proteases transform whey proteins into biopeptides with physiological activities*

• *Lactose can be transformed into prebiotic compounds using ß-galactosidases*

## Introduction

Enzymes are proteins involved in accelerating the biochemical reactions which convert substrates into products. Interest in enzymatic processes is increasing because their biocatalysis is highly active, selective, stable, and environmentally friendly. The global enzyme market size was valued at USD 9008.7 million in 2019 and is expected to grow to USD 13,815.2 million in 2027, exhibiting a compound annual growth rate (CAGR) of 6.4% in the period 2020–2027 (www.fortunebusinessinsights.com). The growth of the enzyme market size is driven by the wide variety of product applications, from food and beverages to detergents, from diagnostics to renewable biofuels and greener production of tires and adhesives (Singhania et al. 2015).

The commercially available enzymes consist mainly of oxidoreductases, transferases, and hydrolases. Hydrolytic enzymes (EC 3.X) are a type of enzyme that break chemical bonds with water, causing a larger molecule to be divided into smaller molecules. They are commonly used for substrate breakdown, degradation of toxins, but can also be used for synthesis of biopolymers, by transferring moieties to other molecules to form more complex compounds. The principal hydrolytic enzymes with commercial application are lipases, proteases, epoxide hydrolases, and nitrile hydrolases. Just proteases account for about 60% of the entire enzyme market (Hasan et al. 2022), but is expected to experience considerable growth in the next few years (http://marketsandmarkets.com).

**Fig. 1:**
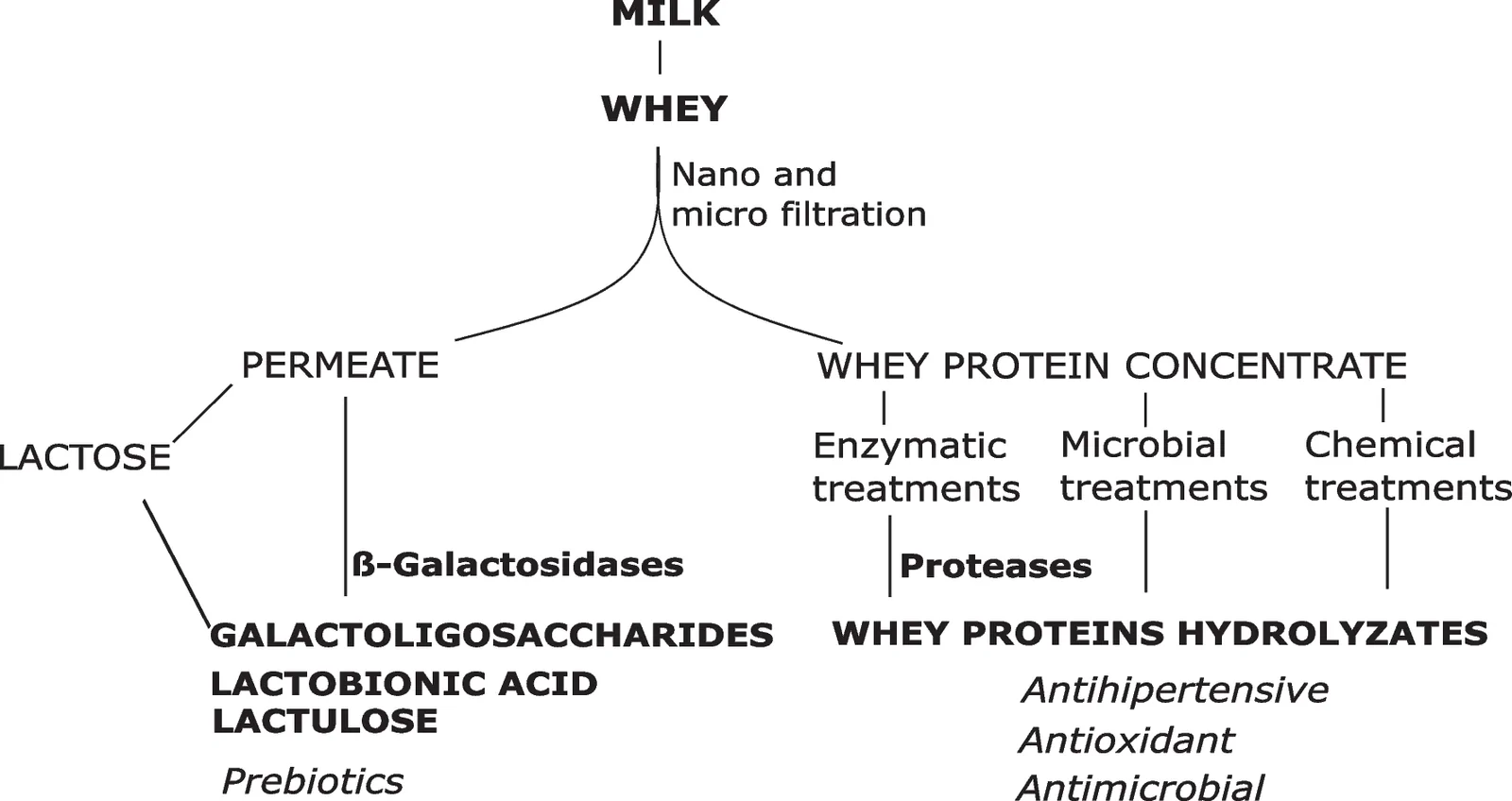
Principal whey components and the main added-value product

One of the areas where enzymatic processes play a key role is whey revalorization. Whey is the main by-product from dairy industries, representing between 85 and 95% of the milk volume used in cheese manufacture and retains around 55% of milk nutrients. It is composed mainly of lactose (4.5–5% w/v), proteins (0.6–0.8% w/v), lipids (0.4–0.5–5% w/v), and mineral salts (8–10% of dried extract) (Zotta et al. 2020). Depending on the casein precipitation method used to coagulate milk, whey can be classified into sweet or sour whey (Zandona et al. 2021). The first one is obtained by treating milk with rennet and has high levels of lactose, fats, and proteins. On the other hand, sour whey is obtained by fermentation or addition acids and contains higher levels of lactic acid, phosphorus, and calcium. Cheese worldwide production is estimated at 22.6 million tons/year, which involves a worldwide whey accumulation around 180–200 million tons/year (Buchanan et al. 2023). Due to its high organic load and high volumes of production, whey poses an environmental risk if it is not properly managed (Zandona et al. 2021). Whey can be used to produce different value-added products: animal feed, whey protein derivatives using membrane technologies, lactose powder via crystallizing, and lactose or protein derivatives by chemical, microbial, or enzymatic processes (Fig. 1). Although 50% of cheese whey is used in food and pharmaceutical formulations, the remaining volume is large enough to be used in other biotechnological processes (Coelho et al. 2021).

In this review, the current state of enzymatic treatments for valorizing whey proteins and lactose from dairy industry by-products is highlighted. We focus on the most common enzymes used for treatments and the characteristics of the products obtained in each case. Since the number of commercial available enzymes and the sources they come from are still limited, we also discuss the potential of metagenomics techniques to identify novel enzymes with desired characteristics and different enzyme engineering techniques as a way to improve known enzymes activity, opening a huge field for future investigations.

## Whey protein hydrolysis

Whey is composed of several different proteins. The most abundant are β-lactoglobulin and ɑ-lactalbumin, representing around 50% and 20% of whey proteins, respectively (Buchanan et al. 2023). Other proteins and peptides that can be found, but represent a smaller fraction of whey, are glycomacropeptide, bovine serum albumin, immunoglobulins, lactoferrin, and lactoperoxidase.

Whey protein concentrates (WPC) and isolates (WPI) can be obtained from whey by combining different filtration strategies (Smithers 2008). Nanofiltration (pore size between 0.1 and 1 nm) helps bring the overall mineral content down, while ultrafiltration (~ 0.5 µm pore size) allows to retain between 35 and 80% of whey proteins, depending on the volume concentration factor applied (Kelly 2019). These processes have been the most widely adopted and cost-effective separation process to obtain WPC since the 1970s. The introduction in the 1990s of a pretreatment, involving cross-flow microfiltration aided in the removal of residual lipids in advance, helps to elevate protein concentration to isolate status (≥ 90% of proteins on a dry basis). Other methods, such as industrial chromatography, can be applied to isolate whey proteins, but are considered expensive and are only used when other potentially cheaper options are less successful.

Even though WPC and WPI are commodities with high value in themselves, breaking down these proteins into peptides can be an interesting approach to add value to these products (Brandelli et al. 2015). Whey protein hydrolysates (WPHs) are used as a food supplement with better digestibility, such as whey protein-based formulas for infants intolerant to cow’s milk protein (Sinha et al. 2007) or for accelerating recovery from exercise induced muscle damage (Buckley et al. 2010). Peptides from whey have also shown several physiological effects on the body, like working as antihypertensive, antioxidant, antithrombotic, opioid, antimicrobial, cytomodulatory, immuno-modulatory, among others (Mann et al. 2019).

While bioactive peptides remain inactive when they are part of the original protein sequence, they can be released by chemical processes, using an alkaline or acidic media. This strategy presents several disadvantages: it is more difficult to control the process to obtain specific products or avoid certain unwanted products, like free amino acids and small peptides with two or more nonpolar residues, which have been linked to bitter taste (Linde et al. 2009). Furthermore, chemical processes can generate hydrolysates with modified amino acids (Ulug et al. 2021). A greener alternative is subcritical water hydrolysis (SCW), a method that uses water at a temperature between 100 and 374 °C and a pressure less than 22 MPa for adopting properties of other no polar solvents such as hexane. This technique is used for bioactive compound extractions from food and for hydrolysis of biopolymers and proteins (Rivas-Vela et al. 2021). Another example of physical hydrolysis is pulsed electric fields, a novel technology able to hydrolysate food proteins in a non-thermal way where a high intensity pulsed electric field is applied. One more promising method is microwave-assisted extractions (MAE) that use electromagnetic radiation frequency higher than 300 MHz. Particularly, microwave-assisted enzymatic hydrolysis assisted in obtaining bioactive peptides from milk protein concentrates (Uluko et al. 2015).

Other novel physicochemical techniques are being explored to aid in the bioactive peptides production. High hydrostatic pressure (HHP) consists in immerse products in water and applied hydrostatic pressures between 100 and 1000 MPa, with or without heat treatment to create protein conformational changes (Naderi et al. 2017). Ultrasound is another technology that looks to affect protein structure, in this case using ultrasonic waves higher than 20 kHz. Finally, Ohmic heating consists of a passing electric current in a uniform way to heat the whey proteins while protecting their nutritional properties (Costa et al. 2018). All these techniques do not fully break the proteins, but significantly decrease the α-helical protein content and significantly increase its β-sheets and β-turns, which facilitates subsequent hydrolysis treatments (Alizadeh and Aliakbarlu 2020).

Fermentation using lactic acid bacteria is another way to hydrolyze whey proteins (Mayta-Apaza et al. 2021). Lactic acid bacteria produce proteolytic enzymatic systems that allow the hydrolysis of large proteins without chemicals involved in the process (García-Cano et al. 2019). However, the hydrolysates can be used by the bacteria as a substrate for strain growth, reducing the amount of product obtained.

Finally, enzymatic hydrolysis is the most common technique to produce bioactive peptides, being proteases (also referred as peptidases) the most widely used for WPH production. They are capable of carrying out specific and selective protein modifications (León-López et al. 2022). However, most of all current enzymatic treatments still have low yield and high costs (Cruz-Casas et al. 2021). At present, there are many commercially available proteases, mainly from animals, plants, and cultivable microorganisms, but there is an ever-growing need for new sources of food grade industrial enzymes, with different or improved activities (Ahmad et al. 2019).

### Animalproteolytic enzymes

The use of animal proteases in food preparation dates back approximately to 6000 BC, where rennet, a preparation of chymosin and pepsin extracted from unweaned ruminants, was used in cheesemaking (Moschopoulou 2011). Currently, those and other gastrointestinal enzymes, like chymotrypsin, have been used to obtain bioactive peptides that have shown physiological benefits (Li et al. 2023a).

Trypsin and pepsin (Table 1) are enzymes that produce bioactive peptides capable of inhibit angiotensin-converting enzyme (ACE), dipeptidyl peptidase IV (DPP-IV), bioactive peptides with antioxidant activity (Power et al. 2014; Silveira et al. 2013), and iron-binding peptides (Kim et al. 2007). In addition, whey protein hydrolysates obtained using trypsin VI from bovine pancreas showed antibacterial activity against two food pathogens: *Listeria monocytogenes* and *Staphylococcus aureus* (Demers-Mathieu et al. 2013). Interestingly, the utilization of pepsin was reported to generate anticarcinogenic bioactive peptides (Cakir and Tunali-Akbay 2021). Chymotrypsin has been used alone or in combination with trypsin to generate bioactive peptides with antimicrobial activity (Demers-Mathieu et al. 2013, Zapata Bustamante et al. 2021) and antioxidant activity (Kleekayai et al. 2020).

**Table 1:** Most common proteases obtained from animal and their products from whey proteins

Enzyme	Source	Peptide activity	Reference
Trypsin	Bovine digestive tract	ACE inhibition, DPP-IV inhibition, antioxidants	Power et al. (2014); Silveira et al. (2013)
		Iron binding	Kim et al. (2007)
		Antimicrobial	Demers-Mathieu et al. (2013)
Pepsin	Bovine digestive tract	ACE inhibition, DPP-IV inhibition, antioxidants	Power et al. (2014); Silveira et al. (2013)
		Iron binding	Kim et al. (2007)
Chymotrypsin	Bovine digestive tract	Antimicrobial	Demers-Mathieu et al. (2013)

### Plantproteolytic enzymes

Plant proteases are still less extensively used than animal sources (Table 2). They show higher proteolytic activity when compared with animal proteases (Feijoo-Siota and Villa 2011), which would make them not suitable to be applied on food products, due to the potential release of free amino acids and small peptides.

**Table 2:** Proteases obtained from plants and their products from whey proteins

Enzyme	Source	Peptide activity	Reference
Papain	Papaya	Antioxidants	Mohan et al. (2015)
	Melon fruit	ACE inhibition	Mazorra-Manzano et al. (2020)
	Trompillo berries	ACE inhibition	Mazorra-Manzano et al. (2020)
	Citrus flowers	ACE inhibition	Mazorra-Manzano et al. (2020)
	Wild thistle	ACE inhibition	Tavares et al. (2011)

Nevertheless, there are cases of plant proteases successfully used to obtain bioactive peptides from whey proteins. Papain, an extensively used cysteine protease, was used to obtain bioactive peptides with antioxidant activity from WPI (Mohan et al. 2015). Also, three novel plant proteases derived from melon fruit, trompillo berries, and citrus flowers were used to generate bioactive peptides with the capacity to inhibit ACE in vitro (Mazorra-Manzano et al. 2020). Previously, Tavares et al. described the optimization of bioactive peptide production based on whey protein using aqueous extracts of *Cynara cardunculus* (Tavares et al. 2011).

### Microbial proteolytic enzymes

Even though animal proteases have been used for centuries to transform whey and other products, proteases from microorganisms have gained more attention over the last years since they offer several advantages over other proteases sources. They can be produced more rapidly and at lower cost than animal enzymes (Putatunda et al. 2019), and show a higher thermostability, since microorganisms can be adapted to a wider range of environments (Bhatia et al. 2021). Moreover, simpler genetic manipulation of microorganisms allowed to obtain enzymes with desired features, like extracellular production (Razzaq et al. 2019) or growth on waste materials (Limkar et al. 2019), obtaining higher productivity with lower production costs (Solanki et al. 2021).

Currently, there are commercial available proteases obtained from microorganisms (Table 3). In particular, the *Bacillus* genus has a very prominent place in terms of the commercial production of proteases, among *Bacteria*. The most common enzymes used belong to the subtilisin-like family, like Alcalase, a serine endopeptidase that consists primarily of subtilisin A from *Bacillus. licheniformis.* It is used for protein hydrolysis in various industrial and research applications. Several reports date the utilization for bioactive peptide production with high antioxidant (de Castro and Sato 2014; Bustamante et al. 2021) and ACE inhibition capacities (Eberhardt et al. 2019). Another example of bacterial proteases is thermolysin, a neutral metalloprotease obtained from *Bacillus stearothermophilus*, which was used to obtain bioactive peptides from α-lactalbumin with antioxidant (Sadat et al. 2011) and ACE-inhibitory activity from β-lactoglobulin (Hernández-Ledesma et al. 2006).

**Table 3:** Commercial available microbial enzymes and their products from whey proteins

Enzyme	Source	Peptide activity	Reference
Alcalase (subtilisin)	*B. licheniformis*	Antioxidant	de Castro and Sato (2014); Bustamante et al. 2021)
		ACE inhibition	Eberhardt et al. (2019)
Thermolysin	*B. stearothermophilus*	Antioxidant	Sadat et al. (2011)
		ACE inhibition	Hernández-Ledesma et al. (2006)
Flavourzyme	*A. oryzae*	Antioxidant, ACE inhibition	Morales García et al. (2021); Ni et al. (2020); de Castro and Sato (2014); Bustamante et al. (2021)

Another important type of microbial proteases extensively used are fungal proteases. They are predominantly produced by members of the genera *Aspergillus*, *Trichoderma*, and *Penicillium* (Gurumallesh et al. 2019). One popular preparation used to modify whey proteins is flavourzyme, a peptidase mixture derived from *Aspergillus oryzae.* This preparation has eight different enzymes identified: two aminopeptidases, two dipeptidyl peptidases, three endopeptidases, and one α-amylase (Merz et al. 2015). It has been used to obtain bioactive peptides with antioxidant (Morales García et al. 2021; Ni et al. 2020; de Castro and Sato 2014; Bustamante et al. 2021) and inhibition of ACE activity.

### Metagenomics proteolytic enzymes

The ever-growing need for novel enzymes and the development of new cheaper sequencing techniques has pushed forward the development of culture-independent techniques, like metagenomics, that allow the study of yet unknown microorganisms. These techniques involve cloning and analyzing DNA extracted directly from environmental samples, opening the door to the identification of novel enzymes from both cultivated and uncultivated microorganisms (Prayogo et al. 2020). There are two main metagenomics strategies for bioprospecting novel enzymes: sequence-based metagenomics and functional or activity-based metagenomics (Escuder-Rodríguez et al. 2018). Both cases start with a DNA extraction from an environmental sample (Costa et al. 2021). Sequence-based strategies involve the whole metagenomic DNA sequencing and identification of candidate genes by comparing predicted genes with sequences of known activity. These genes are then cloned into expression vectors and expressed in suitable hosts. Sequence-based metagenomics allows a deeper study of the microbial community and is not biased toward functional screening methods, but it has a high bioinformatics demand and the quality of the results obtained rely heavily on the databases used for the gene annotation.

On the other hand, functional-based screenings involve fragmentation of total environmental DNA, and cloning those fragments into expression vectors. The obtained clones are then functionally screened for specific activities (Robinson et al. 2021). The main advantage of this approach is that every positive clone is a functional enzyme. However, it has some drawbacks. First of all, it requires the selection of the most suitable expression vector, in which the environmental DNA fragment will be inserted. For small DNA fragments, ≤ 15 kb insert size, plasmids can be used. Some expression vectors, such as fosmids and cosmids (< 40 kb insert size), or bacterial artificial chromosomes (BACs) and yeast artificial chromosomes (YACs) (> 40 kb insert size), allow the recovery of large biosynthetic gene clusters that encode the production of one or more specialized metabolites. Also, selecting the correct expression vector, finding the right expression conditions, having an efficient functional screening method, and avoiding DNA contamination of samples are essential for this approach (Escuder-Rodríguez et al. 2018). Another drawback from functional studies is that they imply the screening of a high number of clones to find the positive ones. However, functional metagenomics ensures that the identified candidates have the expected activity.

Both techniques have been successfully used to identify novel enzymes with promising catalytic activities (Table 4). There have been reports of several serine proteases identified from functional metagenomics: alkaline thermophilic protease (Zhang et al. 2011; Sun et al. 2020; Pessoa et al. 2017), alkaline proteases with high optimal pH (Devi et al. 2016; Neveu et al. 2011; Pushpam et al. 20111), and other mesophilic proteases (Purohit and Singh 2013; Biver et al. 2013). Via sequence-based metagenomics, only a few proteases have been identified to date. Among them, Prt1SU, a mesophilic protease obtained from a solid tannery waste metagenome (Verma and Sharma 2021), PersiProtease1 stable under various harsh conditions from tannery wastewater (Ariaeenejad et al. 2022), and Pr05, Pr06, Pr10 mesophilic proteases from dairy stabilization ponds (Irazoqui et al. 2023). Interestingly, the novel proteases identified by this approach PersiProtease 1, Pr05, Pr06, and Pr10, were able to hydrolyze WPC.

**Table 4:** Examples of metagenomics identified proteases

Enzyme	Source	Approach	Reference
ACPRO001	Antarctic coastal sediment	Functional	Zhang et al. (2011)
pF1AL2	Sea sediment	Functional	Sun et al. (2020)
PR4A3	Mangrove sediment	Functional	Pessoa et al. (2017)
Prt1A	Activated sludge wastewater	Functional	Devi et al. (2016)
M30, DV1	Sand	Functional	Neveu et al. (2011)
AS-protease	Goat skin	Functional	Pushpam et al. (2011)
Alkaline protease	Soil	Functional	Purohit and Singh (2013)
SBcas3	Forest soil	Functional	Biver et al. (2013)
Prt1SU	Solid tannery waste	Sequence based	Verma and Sharma (2021)
PersiProtease1	Tannery wastewater	Sequence based	Ariaeenejad et al. (2022)
Pr05, Pr06, Pr10	Dairy stabilization ponds	Sequence based	Irazoqui et al. (2023)

Finally, protein engineering technologies provide efficient ways to obtain desired characteristics of proteases such as cool adapted activities for food industries (Wang et al. 2023). Two main strategies for protease engineering are commonly used: rational design and directed evolution. Rational design was used to improve thermal stability (Ashraf et al. 2019; Osire et al. 2019) and catalytic performance (Jaouadi et al. 2010) in proteases. On the other hand, directed evolution to increase the activity and organic solvent resistance of a metalloprotease (Zhu et al. 2020) and to improve the proteolytic activity of a *Bacillus pumilus* in lower temperatures (Zhao and Feng 2018) was applied. To the best of our knowledge, no such efforts have been made to improve enzymes used for WPH production.

## Other value-added products from whey: lactose derivatives

Carbohydrate segment held the leading growing market and is anticipated to account for the largest share in the market by 2027. Carbozymes are used in a wide range of applications such as industrial processes and products, prominently in food and beverage industries. In this way, whey also represents an appealing starting point to produce value-added products from milk sugar, lactose.

Lactose is the most important component in whey (4.5–5% w/v) after water (85–95% w/v). It is a disaccharide (β-D-galactopyranosyl-(1 → 4)-D-glucose) which by hydrolysis yields monomers of D-glucose and D-galactose. Commercially, it is one of the major dairy ingredients with numerous applications in different industries, including food industry for manufacturing creams, bakery products, and in the feed industry, predominantly for pig feed. Moreover, it is widely used for infant formula and as excipient or filler in the pharmaceutical industry. The global lactose market was valued at USD 2.22 billion in 2021 and is projected to grow to $3.00 billion by 2029 (www.fortunebusinessinsights.com).

Interestingly, in humans, lactose maldigestion increases with age: there have been reported levels of approximately 70% of the adult population in the world with this condition (Li et al. 2023b). These individuals tend to avoid consuming milk due to the risks of severe abdominal discomfort. In this context, it becomes necessary to transform lactose from whey, chemically, microbiologically, or enzymatically, to prepare derivatives for intolerant individuals and to obtain high value-added products (Rocha and Guerra 2020).

ß-galactosidases (E.C.3.2.1.23) are enzymes capable of hydrolyzing lactose. Firstly, an enzyme-galactose complex is formed and simultaneously the glucose is released. Subsequently, the enzyme transfers the galactose moiety to a nucleophilic acceptor that contains a hydroxyl group. If water is the nucleophilic acceptor, galactose is released and hydrolysis is completed. The mechanism of lactose hydrolysis can also have a transgalactolytic nature: if the galactose is transferred to a sugar, di-, tri-, and oligosaccharides of higher molecular weight are formed (Torres et al. 2010). These galactooligosaccharides (GOS) have been shown to have prebiotic properties, related to their impact on the composition and activities of the intestinal microbiota (Knol et al. 2005). The balance between both activities and the type of GOS obtained are highly dependent on the enzyme, but could be regulated by changing the concentration of galactose donors (Guerrero et al. 2015). Typically, around 30% and 50% of the initial lactose can be converted into GOS, and rarely surpass the 50 g 100 g − 1 levels, for a starting lactose concentration of 270 and 600 g L − 1 (Lamsal 2012). The initial lactose concentration is a key factor for GOS synthesis, as it determines the availability of galactosyl acceptors: more lactose available can increase the rate of GOS production.

The interest in GOS production and application in various food processes and pharmaceuticals has increased significantly (Souza et al. 2022). The market price for GOS is 10 to 12 times higher than that of edible lactose, estimated price around USD 10,000 a ton and world market size is approximately 20,000–22,000 tons/year with the largest annual increase of all lactose derivatives (10 to 20%) (Li et al. 2023c). For example, the addition of GOS in infant foods or foods specialized for the elderly and hospitalized people is a promising field, since these people are more vulnerable to alterations in the gut microbiota (Lamsal 2012).

In terms of the origin of ß-galactosidases, the enzymes are produced mainly by microorganisms, such as filamentous fungi, like *Aspergillus lacticoffeatus* and *Aspergillus oryzae*, bacteria, like *Bifidobacterium longum* and *Lactobacillus delbrueckii*, and yeasts, like strains of *Kluyveromeyces sp* (Souza et al. 2022). As with proteases, there is an ever need for novel enzymes. In this sense, metagenomics also represents an alternative to identify ß-galactosidases active at different environmental conditions and with improved activity (Liu et al. 2019; Eberhardt et al. 2020).

In addition to the search of novel enzymes, multiple efforts have been put in place to improve the enzymatic activity of β-galactosidases, by increasing the transgalactolitic activity, widening substrate specificity, increasing thermal stability, or reducing product inhibition (Movahedpour et al. 2022). A wide array of techniques has been used, such as site-directed mutagenesis, truncation, site-saturation mutagenesis, random mutagenesis, DNA shuffling, and monobody modifications (Lu et al. 2019). Two mutants of a β-glycosidase from *Halothermothrix orenii*, Y296F and F417S, showed an increased GOS production of around 10% (Hassan et al. 2016) and the GOS production of a β‑galactosidase from *Thermotoga maritima* was around 30% to 40% higher when the D568 residue was mutated with a serine and alanine, respectively (Talens-Perales et al. 2016). On the other hand, the substrate repertoire and the transglycosylation kinetics of the *Agrobacterium sp.* β-glycosidase were improved using a random mutagenesis approach (Kim et al. 2004). Another example of protein engineering is the addition of carbohydrate-binding modules (CMB) to the *Thermotoga maritima* β‐galactosidase to improve its immobilization: by adding *Pyrococcus furiosus* chitinase family 2 CBM the authors were capable of achieving nearly 100% of immobilization (Míguez Amil et al. 2019).

Besides GOS, other products can also be obtained from lactose, which have been comprehensively reviewed by many authors (da Silva et al. 2015; Schaafsma 2008). Lactulose (4-o-β-galactopyranosyl-d-fructose) is a lactose isomer with prebiotic properties and broad medical uses, like mildly purgative action and inhibitory properties against ammonia-producing microorganisms (Schaafsma 2008; Zimmer et al. 2017). Lactulose is produced commercially by isomerization (alkali hydroxide catalysis or alkaline epimerization) of α-lactose with calcium hydroxide as the catalyst (Audic et al. 2003), although it can also be obtained using ß-galactosidases to cleave the lactose and having fructose as the nucleophilic acceptor (Ubilla et al. 2020).

Another interesting derivative is lactobionic acid, chemically constituted of a gluconic acid bonded to a galactose. It is known for its numerous proven attributes as an antioxidant, chelator, and moisturizer agent (Cardoso et al. 2019). Lactobionic acid is produced by catalytic oxidation of the aldehyde group from lactose. Recent studies focus on biotechnological processes, using microbial enzymatic routes, to obtain this compound (Alonso et al. 2013; Gupta et al. 2018) but none of them are industrial-scale methods yet.

## Conclusions and future perspectives

Cheese whey is both an environmental concern and an interesting raw material for value-added products due to its high organic load, mainly proteins and lactose. In this sense, there are three main options that can be used to transform these compounds into commodities: chemical, microbial, and enzymatic treatments. Enzymes represent a less polluting, more specific, and safer option, which explains why their market has been growing significantly over the past few years.

Proteases are the enzymes used to hydrolyze whey proteins. These hydrolysates not only have a better digestibility but can also have a large number of physiological effects on the body, making them interesting products. Although there are a great number of commercially available enzymes, the number of species used to obtain them is limited. Nowadays, there are three main sources of proteases: ruminants’ digestive tract, microorganisms, and, to a lesser extent, plants. It is important to notice that, besides plant proteases being more proteolytic than those from animals and microbes, no significant differences were noted between products obtained with enzymes from different sources. The main difference between sources lies in how the enzymes are produced. For centuries, animals were a simple and cheap way to obtain enzymes, but the advancement in biotechnological techniques, such as heterologous expression and protein engineering, allowed the production of new and improved enzymes, with increased catalytic activity or better adapted to different environmental conditions. In this sense, metagenomics has opened the door for a wide array of microbial enzymes with untapped potential. One future perspective involving proteases is the design of enzymatic cocktails, combining enzymes with diverse activity or hydrolysis profiles. These combinations could represent a way to obtain different hydrolysates or to improve process productivity.

Lactose can also be transformed into other value-added products, mainly by the utilization of ß-galactosidases. In contrast with proteases, the modification of lactose requires specific reactions and controlled conditions. The main products obtained from lactose are GOS, used on infant formula and elderly adults’ food since they have prebiotic activity. Lactulose is another interesting product because of its prebiotic properties and broad medical applications, and can be synthesized from lactose using ß-galactosidases. The number of commercially available ß-galactosidases is still low when compared to proteases, being microorganisms the main sources for these enzymes, mostly fungi from the genera *Aspergillus* and *Kluveromyces* and bacteria from the *Bacillus* genus.

One of the most interesting aspects of enzymatic treatments is that they can be applied on a broad range of conditions, since different enzymes can tolerate, for example, different temperatures or pH levels. In this regard, metagenomics proves an appealing tool to identify novel enzymes, from both cultivable and uncultivable microorganisms. Metagenomics screening can provide novel enzymes with different ranges of physicochemical conditions and novel products, to tailor the process to different or more specific conditions and to desired products. In addition, the development of enzyme engineering strategies provides an interesting opportunity to create highly efficient enzymes for the cost-effective production of value-added products.
